# Zoonoses and gold mining: A cross-sectional study to assess yellow fever immunization, Q fever, leptospirosis and leishmaniasis among the population working on illegal mining camps in French Guiana

**DOI:** 10.1371/journal.pntd.0010326

**Published:** 2022-08-15

**Authors:** Maylis Douine, Timothée Bonifay, Yann Lambert, Louise Mutricy, Muriel Suzanne Galindo, Audrey Godin, Pascale Bourhy, Mathieu Picardeau, Mona Saout, Magalie Demar, Alice Sanna, Emilie Mosnier, Romain Blaizot, Pierre Couppié, Mathieu Nacher, Antoine Adenis, Martha Suarez-Mutis, Stephen Vreden, Loïc Epelboin, Roxane Schaub

**Affiliations:** 1 Centre d’Investigation Clinique Antilles-Guyane (Inserm 1424), Centre Hospitalier de Cayenne, Cayenne, French Guiana; 2 TBIP, Université de la Guyane, Cayenne, French Guiana; 3 Centre de Ressources Biologiques Amazonie, Cayenne, French Guiana; 4 National Reference Center for Leptospirosis, Biology of Spirochetes unit, Institut Pasteur, Paris, France; 5 University Laboratory of Mycology-Parasitology, Centre Hospitalier de Cayenne, Cayenne, French Guiana; 6 Delocalized Health Centers, Centre Hospitalier de Cayenne, Cayenne, French Guiana; 7 Department of Dermatology, Centre Hospitalier de Cayenne, Cayenne, French Guiana; 8 Laboratory of Parasitic Diseases, Institute Oswaldo Cruz/Fiocruz, Rio de Janeiro, Brazil; 9 Foundation for Scientific Research Suriname, Paramaribo, Suriname; 10 Infectious and Tropical Diseases Unit, Centre Hospitalier de Cayenne, Cayenne, French Guiana; Faculty of Medicine and Health Sciences, Universiti Putra Malaysia, MALAYSIA

## Abstract

**Background:**

Most emerging pathogens are zoonoses and have a wildlife origin. Anthropization and disruption of ecosystems favor the crossing of inter-species barriers. We hypothesize that the marginalized population of undocumented goldminers in the Amazon is at risk of acquiring zoonoses.

**Method:**

A multicentric cross-sectional study included consenting gold-mining adult workers in 2019. A clinical examination recorded dermatological signs of leishmaniosis and past history of yellow fever vaccination. Biological tests were performed for yellow fever, Q fever and leptospirosis serologies. Additional blood samples from a previous study in 2015 were also tested for leptospirosis.

**Results:**

In 2019, 380 individuals were included in the study, along with 407 samples from the 2015 biological collection. The seroprevalence of leptospirosis was 31.0% [95%CI = 26.4–35.5] in 2015 and 28.1% [23.5–32.7] in 2019. The seroprevalence of Q fever was 2.9% [1.2–4.6]. The majority of participants reported being vaccinated against yellow fever (93.6%) and 97.9% had seroneutralizing antibodies. The prevalence of suspected active mucocutaneous leishmaniasis was 2.4% [0.8–3.9].

**Discussion:**

These unique data shed new light on the transmission cycles of zoonoses still poorly understood in the region. They support the existence of a wild cycle of leptospirosis but not of Q fever. Leishmaniasis prevalence was high because of life conditions and tree felling. High yellow fever vaccine coverage was reassuring in this endemic area. In the era of global health, special attention must be paid to these vulnerable populations in direct contact with the tropical ecosystem and away from the health care system.

## Author summary

Many pathogens are zoonotic in origin and human transmission can occur via vector, direct contact, inhalation, absorption, among other routes enabling human-reservoir interaction. In Amazonia, the cycle of these pathogens (the reservoir, the mode of transmission …) is not always well known. It is important to better understand these cycles in order to evaluate and anticipate the potential risk for human health, both on an individual and collective scale (risk of epidemic). In French Guiana, a French territory located in the Amazon, undocumented gold miners represent several thousand people mainly from Brazil who work in very remote areas in the middle of the rainforest. Documenting several zoonotic diseases among this population living in the middle of biodiversity is very valuable to better understand these cycles but also to assess the impact for their own health and to identify risks for public health. This article provides new data for four zoonoses: Q-fever, leptospirosis, leishmaniasis and yellow fever in this population and discusses the contributions to the understanding of cycles and public health issues.

## Introduction

Since 1940, the emergence of pathogens has significantly increased worldwide [1,2]. Among these pathogens, 60% are from zoonotic origin, mainly from wildlife (72%), and this proportion is still increasing (2). Vector-borne diseases represent 22.8% of the emerging infectious diseases (EID). There are many reasons for these emergences, such as environmental changes that favor the development of vectors and reservoirs, human behavior especially deforestation of tropical forests, the movement of people, animals and their products around the world, the densification of populations or the massive displacements [3]. The anthropization and disruption of ecosystems favor the crossing of inter-species barriers, especially in areas of high biodiversity such as the Amazon basin, where French Guiana is located. In this French overseas territory, the life cycles of multi-host diseases appear to be even more complex than in other Central and South American settings [4]. This suggests that the greater promiscuity between wildlife and humans due to demographic and economic pressures may provide new contexts for the movement and spread of microbes and their hosts [4].

Among the known risk factors associated with EID, several are prominent in the gold mining activity, such as deforestation, which creates breeding areas for vectors, hunting, close contact with wildlife, or poor sanitary conditions [4,5]. Deforestation also causes contact between humans and vectors usually living in the canopy, and ending up on the ground following the felling of trees. In addition, the remoteness of health facilities leads to care seeking delays and thus might increase potential human-to-human transmission and significant morbidity [6]. For example malaria strongly affects the gold mining population due to vector exposure (*Anopheles*) and inadequate or delayed health care seeking behavior, thus increasing the human reservoir of parasites [7,8]. In French Guiana, the estimated 12,000 gold miners working illegally on hundreds of gold mines in the heart of the Amazon forest are mainly Brazilian nationals and frequently travel through the Guiana Shield and Brazil [9]. In addition to the possibility of pathogen emergence and morbidity in this population, this high mobility also poses the risk of pathogen dissemination.

It is therefore very important to assess zoonoses among this population in order to better understand pathogen cycles and to anticipate public health issues. The present study aims to evaluate the epidemiological situation of four zoonoses in the French Guianese context: yellow fever, Q fever, leptospirosis and leishmaniasis.

## Material and methods

### Ethics statement

The protocol was approved by the National Ethics Board of Suriname (CMWO (Commissie voor Mensgebonden Wetenschappelijk Onderzoek), Opinion Number VG 25–17). The authorization of importation of human biological samples was obtained and the biological collection declared to the French Ministry of Education and Research (DC-2014-2160 and DC-2021-4649). The database was anonymized and registered according to the General Data Protection Regulation (GDPR).

A written consent form was collected after information in the participant’s language on the primary objective of the study related to malaria and secondary objectives related to zoonoses with medical examination (leading to referral to care in case of suspected health problem) and the use of the biological samples for further epidemiological investigations (without rendering personal results).

### Type of study

This study is based on a cross-sectional study conducted in 2019 to describe malaria epidemiology among illegal gold miners working in French Guiana. The study design is fully described elsewhere [7,8].

Four zoonoses were selected as secondary objectives because of their interest for public health in the French Guianese context (Table 1).

**Table 1:** Diseases studied with their ecology, epidemiology, public health issue in the Amazon and assessment method in this study.

		Yellow Fever	Q Fever	Leptospirosis	Leishmaniasis
**Pathogens**		Yellow fever virus	*Coxiella burnettii*	*Leptospira* sp.	*Leishmania guyanensis*
					*Leishmania braziliensis*
					*Others*
**Pathogen cycle in the Amazon**	**Reservoir in the Amazon**	Non-human primates	Unclear	Rodents and many mammals described as carriers of *Leptospira* sp. in South America [10,11]	• *L*. *guyanensis*: two-toed sloth (*Choloepus didactylus*)
					• *L*. *braziliensis*: rodents, Didelphidae…
	**Transmission mode**	Vector-borne through *Haemogogus* sp. and *Aedes* sp.	Probable inhalation of contaminated aerosols	Contact of mucous membranes or injured skin with water and soil contaminated with urine of reservoir animals	Vector-borne through *Lutzomyia* sp
**Public health significance of assessment in the gold miners population**		Risk of epidemic in French Guiana due to the proximity of gold miners and non-human primates in the forest and the presence of the vectors if low vaccination coverage	Better understand the mode of transmission and the reservoir by looking at whether people living in constant contact with biodiversity are more affected	• Evaluation of the morbidity linked to leptospirosis among this exposed population	• Pathology complained as a major problem by the concerned population [6]
				• Evaluation of the risk of misdiagnosis of febrile illnesses in this highly malaria-exposed population	• Increased proportion of *L*. *braziliensis* which poses therapeutic challenges
					• Rumors of intense circulation of black market anti-leishmanial drugs known for potentially severe adverse effects
**Outcome**		• vaccination coverage (declared)	Seroprevalence of Q-fever	• Seroprevalence of leptospirosis	• prevalence of active leishmaniasis lesion
		• proportion of the study population being protected by seroneutralizing antibodies		• Identification of the different serovars	• proportion of different *Leishmaniasis species*
					• use of anti-leishmaniasis drugs during the previous year
**Evaluation criteria**	**questionnaire**	• questionnaire about previous yellow fever immunization			• questionnaire about use of antileishmaniosis treatment during the last year
	**medical exam**				• medical examination: active lesions of leishmaniasis (specific training of the investigating physician in the dermatology department of the Cayenne hospital)
	**lab analyses**	• Seroneutralizing antibody test	• Phase I and II IgG using Enzyme-Linked Immunosorbent Assay (ELISA)	• Microscopic Agglutination Test (MAT)	• Swab of the bottom of the lesion in case of active lesion for PCR
			• For phase I antibodies, the sample was considered positive when the serum OD was >10% above the OD cut-off value. For phase II, antibody activities in IU/mL were calculated following maufacturer’s guidelines using a standard curve which was supplied in the kit.	• A positive case was defined as having a positive MAT with a title > = 1/50 (Goris and Hartskeerl 2014)	
			• A seropositive case was defined as having IgG Phase I positive and/or IgG PhII positive	• Description of serovars with titer > = 1/800 due to numerous cross-reactions	
**Study population** **(Samples collected)**		• 380 persons included on the Maroni river in 2019 (376 samples analyzed *)	• 380 persons included on the Maroni river in 2019 (380 samples analyzed)	• 421 persons included on the Maroni river in 2015 (412 samples analyzed)	• 380 persons included on the Maroni river in 2019
				• 380 persons included on the Maroni river in 2019 (378 samples analyzed*)	

* some of the samples were not full enough to make the aliquots needed for these analyses, resulting in a different number of samples than the number of people included

### Population study

**Fig 1:**
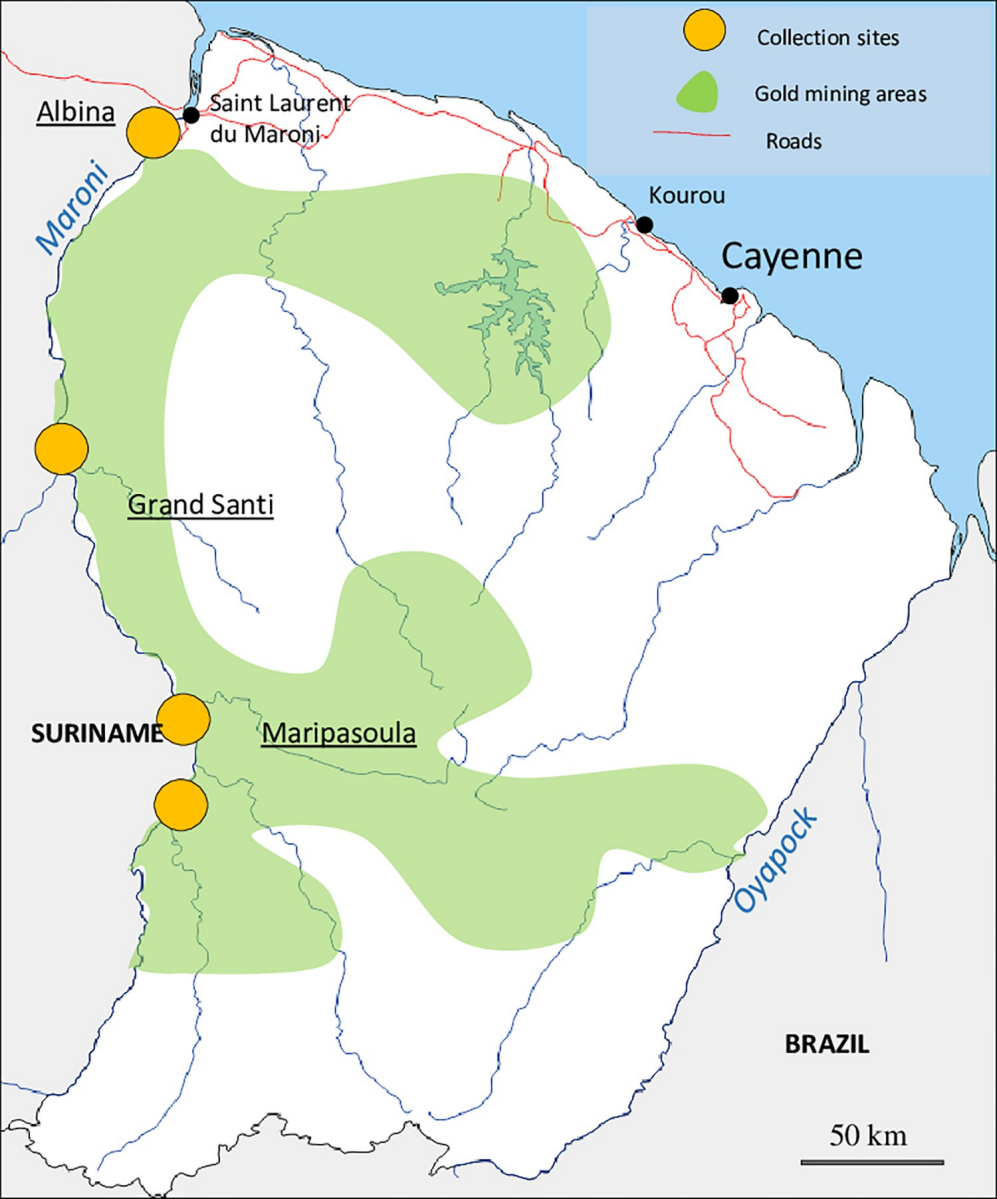
Map of inclusion sites at the Suriname-French Guiana border (map background from dmap https://d-maps.com/pays.php?num_pay=5267&lang=fr).

Participants were enrolled by snow-ball sampling in staging areas, places where gold miners go to sell their gold, buy supplies, rest, or visit family (Fig 1). These places are located along the Maroni River at the border with Suriname. Inclusions were conducted by a trio formed by a health mediator to facilitate communication with the target population, a nurse and a physician.

Inclusion criteria were being over 18 years of age, working at a gold mining site in French Guiana, having left the mine for less than 7 days and agreeing to participate in the study.

### Collected data and evaluation criteria

The cross-sectional survey took place from October to December 2019 (2019 study). Samples from a previous study on similar population in 2015 were added for measuring leptospirosis seroprevalence and its evolution over 4 years. The data presented here are primarily from the 2019-study.

A questionnaire collected sociodemographic information as well as previous yellow fever vaccination (both self-reported and verified by vaccination record if available, number of injections) and use of antileishmanial treatment in the last year and how they obtained it (health facility or self-medication). Medical examination looked for the presence of suspected active mucocutaneous leishmaniasis lesions.

A blood sample was taken at the time of inclusion, centrifuged in the evening, stored at 4°C. The sera were stored at the Biobank Amazonie at -80°C, located in the Cayenne Hospital. The samples were sent afterwards to the different laboratories for further investigation (S1 Circuit: Circuit and pre-analytical conditioning of blood samples). Diagnostic methods are described in Table 1.

### Statistical analyses

An initial analysis described the study population (median with interquartile range or mean with standard deviation according to the variable’s distribution for quantitative variables, frequencies and proportion for qualitative variables) and the prevalence of each zoonosis with 95% confidence intervals. Bivariate analyses assessed the association with potential risk factors, using appropriate tests (chi2 or student’s T tests). Variables with a p-value <0.20 in bivariate analyses were included in a multivariate logistical regression model to assess factors independently associated with leptospirosis. All statistical analyses were performed using Stata 13 (College Station, Texas).

## Results and discussion

### Study population

The characteristics of the 380 individuals included in the 2019 cross-sectional study are described in Table 2. The population included in the 2015 study (N = 421), from which the additional samples tested for leptospirosis came, was comparable and previously described in Douine et al, 2017 [6].

**Table 2:** Characteristic of the study population of 380 clandestine gold miners working in French Guiana, 2019.

			n (%)
**Socio-demographic data**			
	**Age**	Median age [IQR]	39 [31–48]
		< = 29 yo	80 (21.1)
		30–44 yo	168 (44.2)
		> = 45 yo	132 (34.7)
	**Sex**	Women	102 (26.8)
		Men	278 (73.2)
	**Education level**	None or primary	130 (34.3)
		Secondary	239 (63.1)
		Superior	10 (2.6)
	**Country of birth**	Brazil	363 (95.5)
		Other than Brazil	17 (4.5)
**Gold mining activity**			
	**Time in gold mining activity**	≤ 5 years	118 (31.1)
		6–10 years	87 (22.9)
		11–15 years	55 (14.6)
		> 15 years	119 (31.4)
	**Time spent in the last mining site**	< = 6 months	231 (61.8)
		>6months	143 (38.2)
	**Travel time to the actual gold mining site**	<2 hours	29 (7.8)
		2 hours to half day	117 (31.4)
		1 day	92 (24.6)
		> 1 day	98 (26.3)
		Do not know	37 (9.9)
	**Main activity on the mine**	Gold miner	230 (60.4)
		Machine operator	6 (1.6)
		Sex worker	1 (0.3)
		Carrier	16 (4.2)
		Bar owner	4 (1.1)
		Store owner	3 (0.8)
		Housekeeper/cook	54 (14.2)
		Mobile salesman	52 (13.7)
		Unemployed	3 (0.8)
		Transporter	1 (0.3)
		Other	7 (1.8)
		Do not know	3 (0.8)
	**Type of activity**	Non-mobile activity*	305 (80.2)
		Mobile activity**	72 (19.0)
		Do not know	3 (0.8)
	**Number of stays outside from mining site > 3 days during the last year**	Median [IQR]	3 [2–6]

* main activity on the mine itself as gold miner, cook…

** main activity involving great mobility as a transporter, dugout driver…

### Yellow fever

Among the 378 persons who answered the question (two did not know), 93.6% 95%CI [91.2;96.1] reported having received at least one YF vaccine injection. They had received a median of two doses [IQR = 1–2]. YF neutralizing antibodies were found in 368 out of 376 samples analyzed (97.9% 95%CI [96.4;99.3]). Almost all (343/350, 98.0%) of the participants who reported having been vaccinated had indeed protective antibodies; 95.8% (23/24) of those who said not having been vaccinated had, however, protective antibodies. Among the eight persons without YF antibodies, seven declared they were vaccinated. These non-protected persons were young (median = 34.5 YO [28–40]), sex-ratio 1:1, and six with secondary education level.

Despite the existence of an effective vaccine since 1937, yellow fever is responsible for several hundred of cases annually in South America [12,13]. The sylvatic reservoir puts the population living in or near the forest most at risk, as was reported during the large outbreak in Brazil in 2016–2019 [14]. Human-to-human transmission can occur in urban areas due to vector transmission involving *Aedes* sp. The high vaccination coverage of YF in the gold miners’ population (97.9%), above the WHO recommendation of 80%, reflects the effective immunization program in Brazil where the vaccine is free of charge and mandatory for children and recommended in inhabitants of municipalities of the Amazon region, where most miners come from. In the local population of FG, YF vaccine is mandatory, free of charge and readily accessible. Vaccination coverage is estimated at 95.9% 95%CI [95.5;96.3] in primary and secondary school children and 95.0% 95%CI[93.4;96.2] in the general population, but heterogeneous with lower coverage in the vulnerable population (Maroni river region, clandestine immigrants, precarious people…) [15]. The study population appears to be familiar with the YF vaccine as evidenced by the good correlation between reported prior vaccination and the presence of neutralizing antibodies. Although YF vaccination coverage is reassuring in the gold miners’ population, caution is warranted. Indeed, four cases of YF were reported in FG over the past five years, all in unvaccinated people (except one who received one dose in childhood), and all died. Two of them were likely related to gold mining [4,16] (and pers. Com). Physicians should remain vigilant and think of this diagnosis on suggestive symptoms and reported dead monkeys or human cases require a rapid investigation and health response. This surveillance is all the more important as the vaccination schedule has been changed since 2014 from one dose every 10 years to a lifetime dose by the WHO with uncertainties about the persistence of immunity over time [17].

### Q fever

Among the 380 samples, 11 were positive for Q fever (2.9% 95%CI [1.2;4.6]): 6 were positive for both IgG phase I and phase II, 3 for only one IgG and 2 borderline. Among the positive, there were 10 men and one woman, the median age was 45 QIR [34;54] years. Nine were gold miners working directly on the mines (alluvionnary or pits), and they came from six different mining basins widely scattered.

Q fever in French Guiana has a particular epidemiology: the incidence rate is the highest described in the world [18], and its clinical presentation is specific, with more than 25% of community-acquired pneumonia related to *Coxiella burnetii* in French Guiana [19]. If we add to this the potential chronic phase with—among others—cardiovascular disorders, this disease turns into a public health problem in FG. The reservoir is still unknown, despite the incrimination of a species of sloth (*Bradypus tridactylus*) and capybara (*Hydrochoerus hydrochaeris*) from which seropositive samples were found [20]. The seroprevalence of Q-fever among the study population (2.9%) living almost exclusively in the rain forest is lower than that of the general population of FG, estimated at 9.6% in the EPIARBO study (Flamand, C. pers. Com) and up to 20% in the Cayenne urban area and in the agricultural commune of Cacao. In Brazil, the country of origin for most of the gold miners, few data are available showing a seroprevalence of 3.2% among people living with HIV and of 9.3% among intravenous drugs users, which are not comparable to our study population [21,22]. The low seroprevalence in our study does not suggest the existence of a sylvatic reservoir and could be related to reduced presence of livestock in their environment compared to that of the general French Guianese population. Q fever does not seem to constitute a public health issue in this population. Further studies to better understand the reservoir are underway and will shed light on which preventive measures are needed for the most at-risk populations.

### Leptospirosis

The seroprevalence of leptospirosis was measured at 31.0% 95%CI [26.4;35.5] (126/407) in 2015 (5 non-analyzable samples) and 28.1% 95%CI [23.5;32.7] (104/370) in 2019 (8 non-analyzable samples). The antibodies titers were quite low with only six patients presenting titers > = 1/800. Among the latter, serovar analysis shows 2/6 *L*. *interrogans sv Icterohaemorrhagiae* (Table 3).

**Table 3:** Serovars, medical context ang gold mining activity of the six participants with antibodies titers for leptospirosis > = 1/800, French Guiana, 2015 and 2019.

Date	Serovar tested	Titer value	Patient	Gold mining activity*	Medical context at inclusion
**2015**	***L*. *borgpetersenii sv* Castellonis**	1/800	36-year-old man	gold miner in Eau-Claire region, working for 1 year in gold mining	No fever or other clinical signs, malaria RDT and PCR negative
	***L*. *kirschneri sv* Grippotyphosa**	1/800	31-year-old man	gold miner in Mana region, working for 2 years in gold mining	No fever or other clinical signs, malaria RDT and PCR negative
	***L*. *interrogans sv* Icterohaemorrhagiae**	1/800	28-year-old man	dugout driver in Sophia region, working for 6 months in gold mining	Apyretic, asymptomatic, but RDT positive for *P*. *falciparum* confirmed in PCR
	***L*. *interrogans sv* Icterohaemorrhagiae**	1/800	27-year-old man	gold miner in Mana region, working for 10 years in gold mining	No fever or other clinical signs, malaria RDT and PCR negative
	***L*. *noguchi sv* Panama**	1/800	31-year-old man	gold miner in Eau-Claire region, working for 3 years in gold mining	Presented fever, no other symptoms recorded, positive RDT for *P*. *vivax* without PCR confirmation
**2019**	***L*. *kirschneri sv* Grippotyphosa**	1/800	29-year-old man	carrier in Eau-Claire region, working for 10 years in gold mining	Declared having had fever and headache 7 days before, self-medicated with Artemether-Lumefantrine (4 tablets on three occasions), no longer had headache or fever at the time of inclusion but had digestive disorders. Negative RDT and PCR for malaria, was referred to the health center for further investigations (did not go)

* gold miner means person who worked directly on the mine and not for support (logistic, transport, mechanic…)

The seroprevalence was higher in men than in women. Although not significant, it appears that leptospirosis affects mainly men working directly in the mines, especially alluvial mines (Table 4). In univariate analysis, the northwestern part of FG seems to be more affected, but the high mobility of the gold miners prevents conclusions from being drawn.

**Table 4:** Factors associated with positive leptospirosis serology among the population of illegal gold miners included in the 2015 and 2019 studies (univariate and multivariate analysis).

			2015 N = 407				2019 N = 370			
			n (%)	OR*	p	AOR** N = 363	n (%)	OR*	p	AOR** N = 364
**Socio-demographic data**			* *	* *	* *	* *	* *	* *	* *	* *
* *	**Age**	< = 29 yo	24 (27.3)	/	0.300	/	24 (31.2)	/	0.770	/
		30–44 yo	61 (29.5)	1.1 [0.6–1.9]		1.2 [0.7–2.3]	44 (26.7)	0.8 [0.4–1.5]		0.7 [0.4–1.4]
		> = 45 yo	41 (36.6)	1.5 [0.8–2.8]		1.7 [0.8–3.3]	36 (28.1)	0.9 [4.6–1.6]		0.7 [0.4–1.3]
* *	**Sex**	Women	24 (20.2)	/	**0.002**	/	13 (13.0)	/	**<0,001**	/
		Men	102 (35.4)	2.2 [1.3–3.6]		2.5 [1.4–4.5]	91 (33.7)	3.4 [1.8–6.4]		3.3 [1.7–6.5]
* *	**Education level**	None or primary	66 (33.7)	/	0.254	* *	38 (30.2)	/	0.545	* *
		Secondary or superior	60 (28.4)	0.8 [0.5–1.2]		* *	66 (27.2)	0.9 [0.5–1.4]		* *
* *	**Country of birth**	Other than Brazil	6 (24.0)	/	0.427	* *	1 (5.9)	/	**0.017**	* *
		Brazil	120 (31.4]	1.5 [0.6–3.7]		* *	103 (29.2)	6.6 [0.9–50.4]		* *
**Gold mining activity**			* *	* *	* *	* *	* *	* *	* *	* *
* *	**Time in gold mining activity**	< = 10 years	67 (27.9)	/	**0.113**	* *	54 (27.0)	/	0.671	* *
		>10 years	59 (35.3)	1.0 [1.0–1.0]		* *	49 (29.0)	1.0 [1.0–1.0]		* *
* *	**Time spent in the last mining site**	< = 6 months	61 (29.8)	/	0.679	* *	53 (23.5)	/	**0.014**	/
* *		> 6 months	63 (31.7)	1.0 [1.0–1.0]		* *	49 35.5]	1.0 [1.0–1.1]		1.0 [1.0–1.1]
* *	**Travel time to the actual gold mining site**	< = 1 day	38 (27.7)	/	0.373	* *	44 (30.8)	/	0.411	
		> 1 day	78 (32.1)	1.2 [0.8–2.0]		* *	49 (26.6)	0.8 [0.5–1.3]		* *
* *	**Type of activity**	Other than gold miners	55 (26.7)	/	**0,159**	* *	28 (18.9)	/	**0,001**	* *
* *		Gold miner	71 (35.3)	1.5 [1.0–2.3]		* *	76 (34.2)	2.2 [1.4–3.7]		* *
* *	**Type of gold mine**	Pits	5 (15.2)	/	**0.055**	* *	22 (31.4)	/	0.511	* *
* *		Alluvionnary	59 (30.0)	2.4 [0.9–6.5]		* *	67 (27.7)	0.8 [0.5–1.5]		* *
* *		Both	56 (35.2)	3.0 [1.1–8.3]		* *	9 (21.4)	0.6 [0.2–1.5]		* *
* *	**Pace of work**	Day	103 (32.4)	/	**0.178**	* *	76 (27.7)	/	0.638	* *
* *		Night or both	22 (25.0)	0.7 [0.4–1.2]		* *	27 (30.3)	1.1 [0.7–1.9]		* *
* *	**Place of work**	North-West	40 (38.8)	/	**0.156**	* *	45 (32.1)	/	0.493	* *
* *		South-West	75 (29.0)	0.6 [0.4–1.0]		* *	55 (26.3)	0.8 [0.5–1.2]		* *
* *		South-East	3 (17.6)	0.3 [0.1–1.2]		* *	1 (33.3)	1.1 [0.1–11.9]		* *
* *		North-East	1 (50.0)	1.6 [0.9–25.9]		* *	0 (0)	/	* *	* *
* *	**Number of mines worked in during the last 3 years**	< = 3	75 (29.8)	/	0.506	* *	66 (24.9)	/	**0.032**	/
* *		>3	51 (32.9)	1.0 [1.0–1.0]		* *	38 (36.2)	1.0 [1.0–1.0]		1.0 [1.0–1.1]

* Odds-Ratio

** Adjusted Odds-Ratio after logistical regression

The seroprevalence of leptospirosis is very high among this population of gold miners (31.0% and 28.1%), when compared to groups with known occupational risk like slaughterhouse workers in New-Zealand (seroprevalence = 10–31% [23]), sewer workers in India (16.6% [24]), or urban cleaners in Paraguay (8.6% (IgG) [25]). In French Guiana, leptospirosis has long been considered as anecdotal but the incidence increased since 2012 with the availability of better diagnosis tools like PCR and IgM serology, until reaching one of the highest in the world [26,27]. A high proportion (25%) of 48 leptospirosis infections diagnosed between 2007 and 2014 were related to gold mining activities [28]. In Brazil, incidence data show that the states of Acre and Amapa have the highest incidences (30.7/100,000 inhab.and 11.7/100,000 inhab.) [29]. The majority of the gold miners come from the States of Maranhão and Pará were the incidences are of 0.4 and 1.8/100,000 inhab. respectively. The duration of antibodies against leptospirosis in the blood persists from a few months for IgM to several years for IgG [30]. Therefore, it is indeed difficult to conclude where the subjects of the study were exposed. However, in South America, many mammals have been identified as *Leptospira* reservoirs [10,11]. The *Leptospira* has been shown to be adapted to tropical climates and aquatic environment such as that of the gold miners [31]. It is therefore possible that there is wild cycle of leptospirosis in Amazonian ecosystems. The symptoms of leptospirosis and malaria–which is highly prevalent in the gold miner population—can be similar, especially at the onset of the disease, thus acute leptospirosis might be underdiagnosed in these population who used to use self-medication with antimalarials antibiotics bought on the black market [32,33]. But using the wrong medication can worsen the health condition and even lead to death, although doxycycline maybe useful in both infections. An innovative health intervention implemented in the region (Malakit) aims to train gold miners to self-diagnose and self-treat malaria in case of symptoms [8]. The training provided in by Malakit should emphasize differential diagnoses and therefore the importance of having an accurate diagnosis before self-medicating.

### Mucocutaneous leishmaniasis

**Fig 2:**
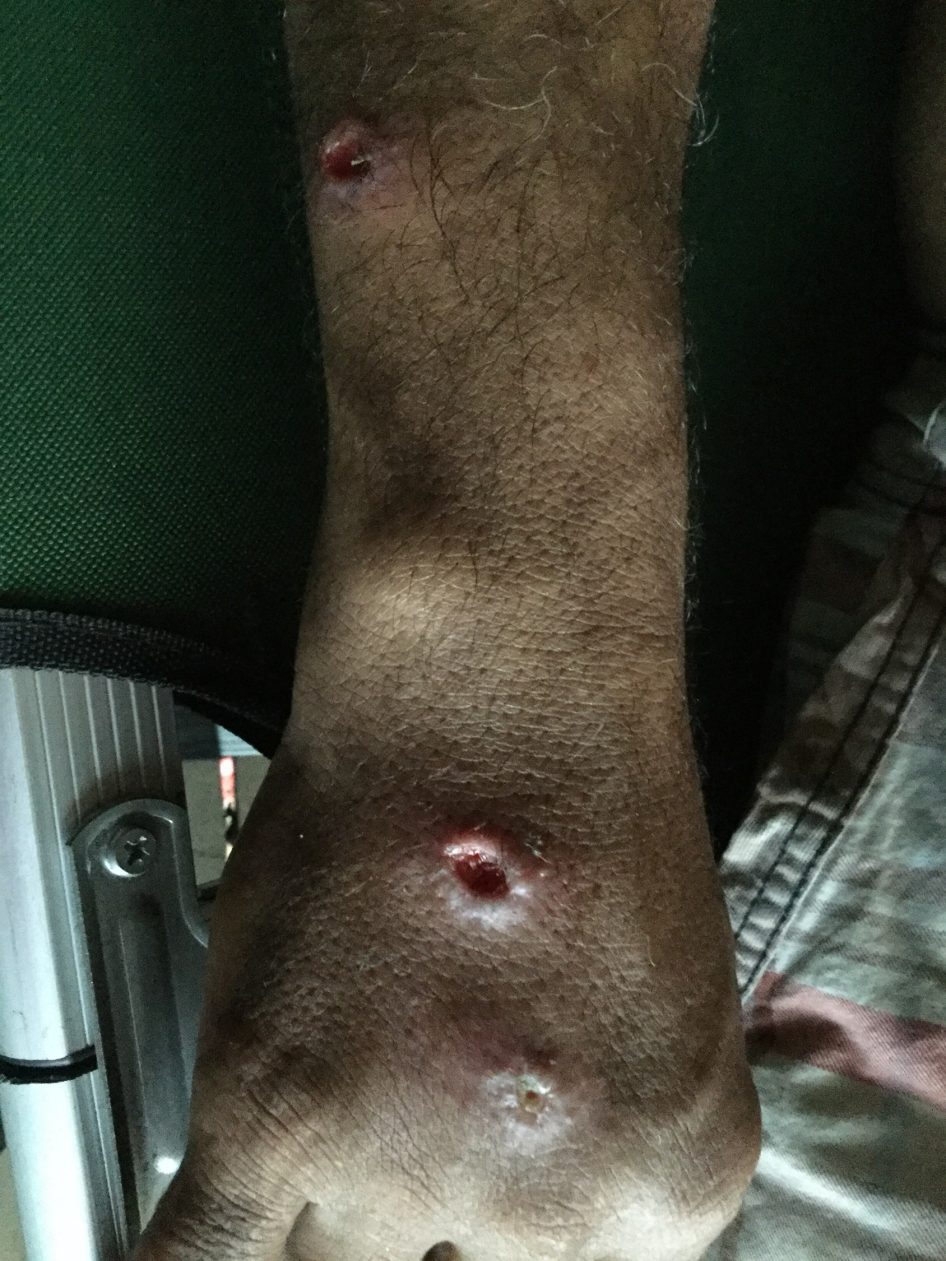
Three cutaneous leishmaniasis lesions on a gold miner included in the 2019 study.

Among the 380 persons included in the study, nine had typical active cutaneous leishmaniasis lesions, so 2.4% 95%CI [0.8;3.9]. The M/F sex ratio was 3.5 (7/2), median age of 41 IQR [36;53] years. They were all Brazilians and they all came from mining camps located in the southwestern part of FG (municipality of Maripasoula) except one from a northwestern mine. Four participants had only one lesion, four had two, and one had four. Five were nodules, three ulcerations (Fig 2) and one non-specified.

Among the three samples of the ulcerated lesions for PCR, one was negative, one was positive for *L*. *guyanensis*, and one was positive without enough amplification to identify the infecting species. Among these nine persons with active leishmaniosis lesions, five reported to have used a pentamidine injection at least once (up to four times) during the previous year: three got pentamidine on the black market, one went to a health center, one first went to a health center and then bought a second dose on the black market. One person self-medicated with the application of sugar with copaiba oil (*Copaifera officinalis*). Among the 362 persons without active leishmaniasis who answered the question, 17 declared having used anti-leishmanial treatment during the previous year (Table 5). The average price for under-the-counter pentamidine was 8 grams of gold for one injection–about 200 USD on gold mines.

**Table 5:** Use of anti-leishmanial treatment in the previous year among gold miners included in the 2019 study along the Maroni border.

		Active lesion of leishmaniasis	No active lesion of leishmaniasis
**N (%)**		9 (2.4)	371 (97.6%)
**Use of antileishmanial treatment during the previous year**			
** **	yes	5 (55.6)	17 (4.6)
** **	no	4 (44.4)	345 (93.0)
** **	missing data	0 (0)	9 (2.4)
**Drug used**			
** **	meglumine antimoniate	0	1
** **	pentamidine	5	16
**Place were the drug was obtained**			
** **	mining site	3 (60.0)	8 (47.1)
** **	health care facility	1 (20.0)	6 (35.3)
** **	both	1 (20.0)	2 (11.7)
** **	do not know	0 (0)	1 (5.9)
**Number of injections during the previous year**			
** **	1	2	3
** **	2		4
** **	3	2	3
** **	4	1	3
** **	5		1
** **	6		1
** **	not specified		2

The prevalence seemed low compared with the 8.3% 95%CI[5.8;11.4] in 2015 along the Maroni river or 4.5% 95%CI[1.4;7.6] and 7.8 95%CI[2.8;12.7] on the Oyapock River in 2018 and 2019 respectively among the same population [6](pers. Com). The study was implemented during the dry season (October to December) in a particularly dry year (2019) which can underestimate the prevalence. However, this remains much higher than in the general French Guianese population (prevalence of 0.1% (pers. Com)) or than in Amerindian population (9/639 = 1,4% [34]). Leishmaniasis is considered one of the main health problems of gold miners, who represent a high proportion of newly diagnosed cases at the hospital in Cayenne [6,35,36]. Our study raised the issue of frequent use of black market medicine with potentially serious side effects (hematological, metabolic and nervous system disorders…). Another issue that needs to be monitored is the increased proportion of *L*. *braziliensis* compared to *L*. *guyanensis* since the 2000s, especially near gold mining areas [37]. This may be related to a different ecosystem for this pathogen whose reservoir would not be the two-toed sloth like *L*. *guyanensis*, or with the advent of PCR which allows diagnostic improvements. Its morbidity is higher with mucocutaneous lesions requiring a five-day treatment with IV liposomal amphotericin B or three weeks of hospital treatment with meglumine antimoniate which is much more expensive.

### Limitations

This study is the first to assess these neglected zoonotic diseases in a hard-to-reach population that is particularly at risk due to its close contact with biodiversity, poor sanitary conditions and remoteness from health facilities. However, some limitations must be taken into account when interpreting the results. The persistence of antibodies over time does not allow for a clear conclusion on the location of infection of the study subjects. The data presented concern participants included along the Maroni River and therefore do not represent the entire population of gold miners in FG. The sensitivity and specificity of the biological tests used as well as the subjectivity of the clinical examination may have led to classification bias. Seasonal variations and outbreaks may not be captured by the cross-sectional design based on seroprevalence while the incidence may vary over time. Finally, as the study was initially designed to study malaria, some outcomes have a large margin of error due to small sample size. Nevertheless, this is the first study to target this sentinel population for emerging diseases and seroprevalences allowed to get a great view of the year-round epidemiological situation.

## Conclusion

While the results are reassuring regarding the risk of a yellow fever or Q fever epidemic, the high seroprevalence of leptospirosis and prevalence of clinical leishmaniasis in this vulnerable and remote population are very concerning. Although the place of contamination cannot be known, this raises the possibility of an increased risk of some anthropo-zoonotic and vector-borne infections in the context of the small scale gold-mining in the Amazon forest. It is therefore important to carefully monitor the health status of this specific population. Other pathogens should be evaluated such as *Toxoplasma gondii*, *Trypanosoma cruzi*, or digestive parasites. In order to be able to identify emerging pathogens, it could be interesting to put in place exploratory studies such as metagenomics. For example, we have described a new species of *Anaplasma*, *A. sparouinense*, in a gold miner by looking for tick-borne diseases in forest workers [38]. The high morbidity to these pathogens, as well as other health disorder (STIs, malnutrition…) among the marginalized population of gold miners is evidence of a health inequity in this European territory of the Amazon (6). It also raises a public health concern because of the potential impact on the general population of French Guiana but also of the Guiana Shield because of the high mobility of gold miners. In the era of Global Health, special attention must be paid to these vulnerable populations in direct contact with the tropical ecosystem far from the care system and therefore under the radar of the usual surveillance system.

## Supporting information

S1 Circuit Circuit and pre-analytical conditioning of blood samples. (DOCX)

S1 Dataset Anonymized data set. (XLSX)
