# Supplementary material for: Zoonoses and gold mining: A cross-sectional study to assess yellow fever immunization, Q fever, leptospirosis and leishmaniasis among the population working on illegal mining camps in French Guiana
Source: PLoS Negl Trop Dis. 2022 Aug 15;16(8):e0010326. doi: 10.1371/journal.pntd.0010326 (PMC9410546; doi:10.1371/journal.pntd.0010326)
Supplement: S1 Circuit — (DOCX) [file pntd.0010326.s001.docx]

Suppl Material

Circuit and pre-analytical conditioning of blood samples

|  | Yellow Fever | Q fever | Leptospirosis | Leishmaniasis |
| --- | --- | --- | --- | --- |
| Type of analyses | Seroneutralizing antibody test | Phase I and II IgG using Enzyme-Linked Immunosorbent Assay (ELISA), Commercial Kit (SERION/VIRION Co., Germany, Kit number ESR 1312 G) according to manufacturer’s instructions | Microscopic Agglutination Test (MAT) | PCR |
| Lab of analyses | Cerba laboratory | University Laboratory of Mycology-Parasitology, Cayenne Hospital | National Reference Center for leptospirosis, Institut Pasteur Paris | University Laboratory of Mycology-Parasitology, Cayenne Hospital |
| Place | Cergy-Pontoise, France | Cayenne, French Guiana | Paris, France | Cayenne, French Guiana |
| Method of sending the samples | Frozen shipment in dry ice by specialized carrier | Direct transmission of samples from the biobank Amazonie to the laboratory | Frozen shipment in dry ice by specialized carrier | Direct transmission of samples from the biobank Amazonie to the laboratory |
